# Proposal of a specific classification of primary periphyseal stress injuries in adolescent rock climbers

**DOI:** 10.3389/fspor.2025.1596624

**Published:** 2025-08-14

**Authors:** V. Schöffl, X. Iruretagoiena, T. Nelson, Paulo Miro

**Affiliations:** ^1^ Department of Orthopedic and Trauma Surgery, Klinikum Bamberg, Bamberg, Germany; ^2^ Department of Trauma Surgery, Friedrich Alexander University of Erlangen–Nuremberg, Erlangen, Germany; ^3^ Section of Wilderness Medicine, Department of Emergency Medicine, University of Colorado School of Medicine, Denver, CO, United States; ^4^ School of Clinical and Applied Sciences, Leeds Becket University, Leeds, United Kingdom; ^5^ Division of Exercise Physiology and Metabolism, Department of Sport Science, University of Bayreuth, Bayreuth, Germany; ^6^ Deusto Physical TherapIker, Physical Therapy Department, Faculty of Health Sciences, University of Deusto, San Sebastian, Spain; ^7^ Camp4 Human Performance, Salt Lake City, UT, United States; ^8^ Department of Radiology and Imaging Sciences, School of Medicine, The University of Utah, Salt Lake City, UT, United States

**Keywords:** rock climbing, epiphyseal fractures, finger injury, fracture classification, periphyseal fractures

## Abstract

**Introduction:**

Primary Periphyseal Stress Injuries (PPSI) of the hand and fingers are relatively uncommon but are most frequently seen in adolescent rock climbers. A major limitation in the current literature on PPSIs is the lack of a standardized nomenclature and radiological classification. This gap complicates the accurate diagnosis, treatment, and comparison of outcomes across studies.

**Methods:**

We conducted a comprehensive structured literature review of the relevant PPSI literature in climbers using Pubmed, SPORTDiscus, and Web of Science to identify the relevant studies on PPSI in adolescent rock climbers. Based on the findings from existing research and our own data, we propose a new classification system for these injuries.

**Results:**

A five-grade classification system, with subgroups, has been developed based on both clinical and radiographic data. The classification is presented in a table, along with figures illustrating examples of the various injury types.

**Conclusion:**

Additional research is required to assess the reliability and reproducibility of this classification system. We plan to conduct these evaluations in future studies.

## Introduction

Primary Periphyseal Stress Injuries (PPSI) of the hand and fingers are relatively rare but are most commonly observed in adolescent rock climbers (1–3). In fact, they are the most common sport-specific injury in young climbers (2, 4) These injuries are rarely the result of a single traumatic event; rather, they are considered chronic injuries (stress fractures) cause by the repetitive, often supra-physiologic stress applied to the fingers during climbing (5). Although PPSIs are by far most commonly associated with rock climbing, they have also been reported, albeit infrequently, in other athletes such as gymnasts, baseball players, and pianists (4).

Physeal stress injuries generally occur when the extremity is subjected to repetitive loading without adequate rest periods to allow for structural adaptation (6–8). Physeal stress injuries affecting the epiphyseal growth plate complex are referred as (1, 4, 8, 9) primary periphyseal stress injuries (6). A recent framework proposed by Caine et al. (6) provides a novel understanding of the pathophysiological mechanisms and outcomes of PPSIs. Diagnosing these injuries in climbers' fingers can be particularly challenging, as they are often not visible on radiographs (9, 10). Additionally, MRI diagnosis can be difficult because of the need for thin-slice, angulated imaging planes (9, 11). Diagnostic and therapeutic guidelines have recently been proposed to address these challenges (10).

One persistent issue highlighted in the literature on PPSIs, both in general and specifically among climbers, is the lack of standardized nomenclature and radiological classification (1, 6, 8, 9). In a recent publication, Caine et al. (6) noted significant inconsistencies and imprecision in the terminology used to describe these injuries. Existing classification systems, such as the Salter-Harris (12) and Aitkens (13) classifications, are widely recognized but lack specificity regarding the pathophysiology of PPSIs (8). The Salter-Harris classification, originally designed for classifying acute fractures involving the growth plate, has been applied to categorize metaphyseal stress injuries in young athletes (6), including climbers' fingers injuries (4, 8, 9). However, while the radiographic appearance of some of these injuries may resemble Salter–Harris type I fractures, the pathology and mechanism of these injuries differ substantially (6, 8). Early-stage stress fractures, which may not show a distinct fracture line on radiographs but exhibit edema on MRI are not represented in the existing classifications for acute fractures. Additionally, dorsal physeal widening seen in stress fractures (radiographic sign 1) is completely different from epiphysiolysis described in Salter-Harris 1. Moreover, the extent of sclerosis in PPSIs cannot be observed in acute fractures and cannot be classified using Salter-Harris or Aitkins. Given these limitations, there is a clear need for the development and validation of a more precise imaging-based classification system for PPSIs in general, as well as one specifically tailored to finger injuries in climbers. Such a system would improve diagnostic consistency and provide better guidance for treatment.

## Methods

Our primary area of research and expertise focuses on climbing-related injuries, including their diagnosis and classification. To inform our study, we performed a structured literature review using Pubmed, SPORTDiscus, and Web of Science–with the final search on March 1st, 2025. This search was supplemented by manually reviewing the reference lists of selected articles to identify additional relevant studies. We employed a combination of MeSH terms and tailored search keywords, including “epiphyseal fractures”, “adolescent climbers”, “finger injuries”, and “youth climbers”.

We reviewed the extracted studies on PPSIs in climbers' fingers (1–5, 9–11, 14–38) and developed a new classification system. This system integrates clinical presentation (4, 5, 10, 16), biomechanics (5, 14), imaging findings (9, 11, 15), and elements from previously established frameworks such as Salter-Harris (12) and Aitkens (13). Additionally, the extent of sclerosis in the fracture line, as observed in CT scans, was considered, as this is a critical variable in the decision algorithm of Schöffl et al. (10) to determine whether surgical spot drilling should be recommended.

## Results

A total of 50 important publications were gathered, and the injuries were analysed with a focus on their classification. Most of what is known about PPSIs among climbers arises from case reports and case series (8). Caine et al. (1) reported in 2021 that overall, there were 11 published case reports and series describing a total of 80 patients, including 65 males and 15 females, between ages 11 and 17, with PPSIs involving the hand and fingers (5, 11, 20–24, 39–43). With newer reports from Schöffl et al. (10), who reported an additional 37 digital PPSIs in 27 patients (19 male, 8 female), there are presently 107 (84 males, 23 females) published cases, making the fingers the most frequent anatomical site for published case reports of PPSIs.

Overall, physeal stress injuries occur when repetitive loading of the extremity is imposed without sufficient interval of rest to allow for structural adaptation (6–8). Physeal stress injuries involving the epiphyseal growth plate complex have been referred as *primary periphyseal stress injuries* (PPSIs) (1, 6). The most frequent digital PPSI reported were Salter-Harris type III involving the dorsal aspect of the middle phalanx, but Salter-Harris type I, II and V have also been reported (3, 5, 8, 11, 16–24, 39–41, 43, 44).

Conventional radiography serves as the primary imaging modality for assessing PPSIs of the fingers, due to its accessibility and cost-effectiveness (9). The most common radiographic manifestation of finger PPSIs is a Salter Harris type III fracture of the dorsal long finger middle phalanx (9, 10). It is recommended to obtain a minimum of two orthogonal views, typically anteroposterior (AP) and lateral views (9). Computed Tomography (CT) provides a more comprehensive assessment of the physis and adjacent osseous structures compared to radiographs (9). CT may reveal radiographically occult periphyseal sclerosis or osteopenia and premature physeal closure (9, 16). Magnetic Resonance Imaging (MRI) presents numerous clinically significant advantages over radiography and CT (4, 9). The most common presentation in MRI is a Salter Harris III fracture, characterized by physeal widening with a fracture through the physeal hyaline cartilage extending to the epiphysis, manifesting as increased T2 or short inversion time inversion-recovery (STIR) signal in these regions (9, 13). Also Salter Harris II and I injuries are reported in MRI (1, 4, 9, 10). Overuse injuries without associated fracture demonstrate similar widening of the physis, but without fracture of the hyaline cartilage, allowing differentiation from Salter-Harris I fractures (16).

In a recent publication Caine et al. (6) discussed inconsistencies and imprecision in the nomenclature used to describe primary periphyseal stress injuries. The use of the Salter-Harris (12) and Aitkens classifications (13, 45), in particular, seem not very specific to the pathophysiology. Initially intended for classifying direct or acute fractures involving the growth plate, the Salter-Harris classification has often been applied in an attempt to categorize metaphyseal stress injuries in young athletes (6, 24, 41, 42). However, while the radiographic appearance of these injuries may appear similar to Salter–Harris type I fractures, the nature and mechanism of the injury are actually quite different (8, 29). The authors conclude that given the short-comings of the Salter–Harris classification for describing these injuries, it follows that there is a need for the future development and testing of a more precise imaging-based classification to grade PPSIs that can be used to guide appropriate treatment (8, 29).

**Figure 1:**
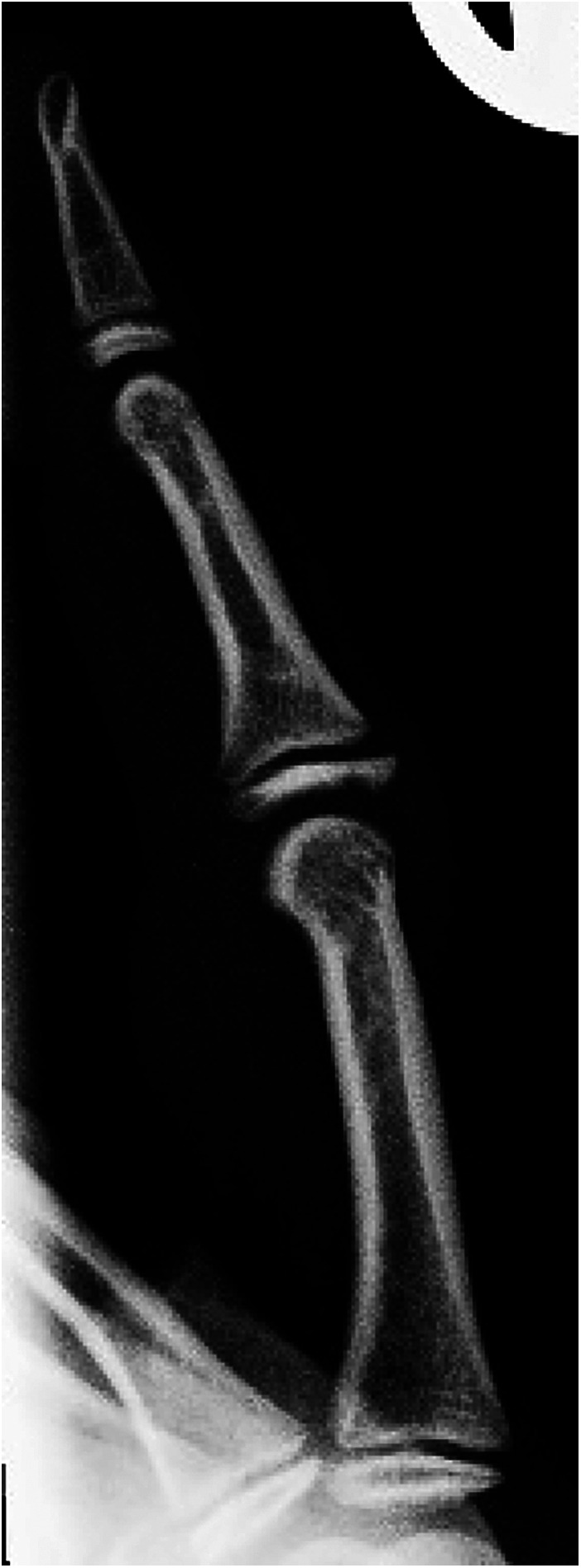
Radiographic sign 1. Dorsal widening of the dorsal middle phalangeal physis with irregularity, fragmentation, and periphyseal osteopenia (4, 8, 9). Physeal widening correlates with hypertrophied chondrocytes extending into the metaphysis, a consequence of disrupted metaphyseal vascular supply (46). Physeal irregularity and fragmentation result, in part, from damage and effacement of the zone of provisional calcification (47). (14 y old girl, right middle finger).

**Figure 2:**
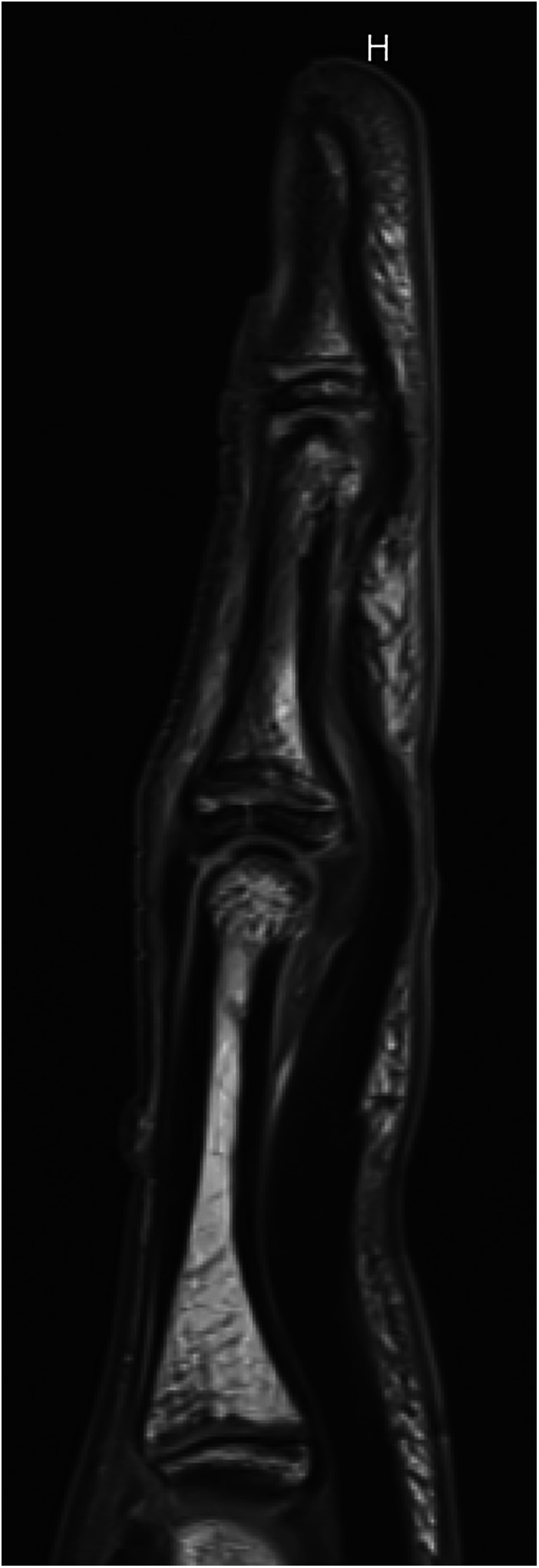
Radiographic sign 2. Non-displaced dorsal fracture of the epiphyseal-metaphyseal-complex (EPM) of the middle phalanx base (5, 9). (13 y old boy, left middle finger).

**Figure 3:**
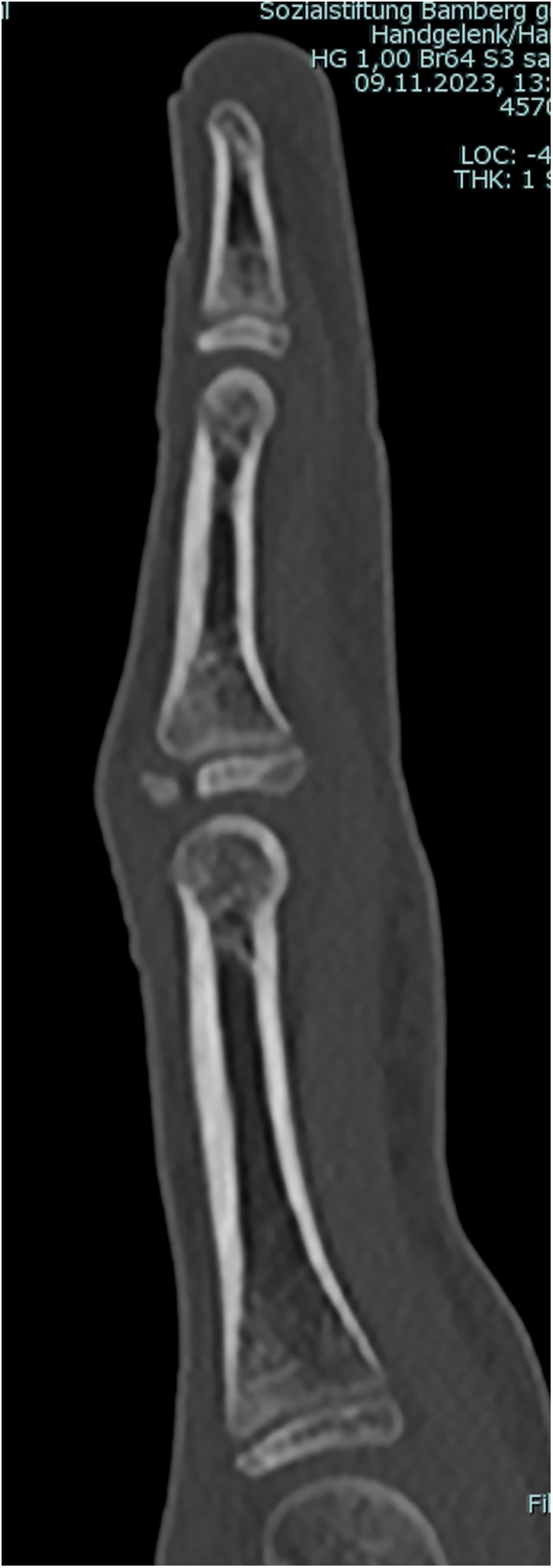
Radiographic sign 3. Displaced dorsal fracture of the epiphyseal-metaphyseal-complex (EPM) of the middle phalanx base (15 y old boy, right middle finger).

The first author has extensively studied the pathophysiology and therapy of these PPSI injuries and the Sportsmedical Center of the Klinikum Bamberg, Bamberg, Germany serves as an international refferal center for these injuries. Thus, based on the analysis of 50 relevant publications, along with our clinical and scientific experience with these fractures, we propose the following classification. The aim of this classification is to combine clinical symptoms with radiological presentations in conventional radiographs, CT scans or MRIs (Figures 1–3). It is important to differentiate between fractures with and without sclerosis on a CT scan, as the presence of sclerosis is an indication for surgery in the Schöffl et al. algorithm (10).

## Proposed classification

### Clinical and radiological classification of primary periphyseal stress injuries in adolescent rock climbers

(see Figures 1–4.)

**Figure 4:**
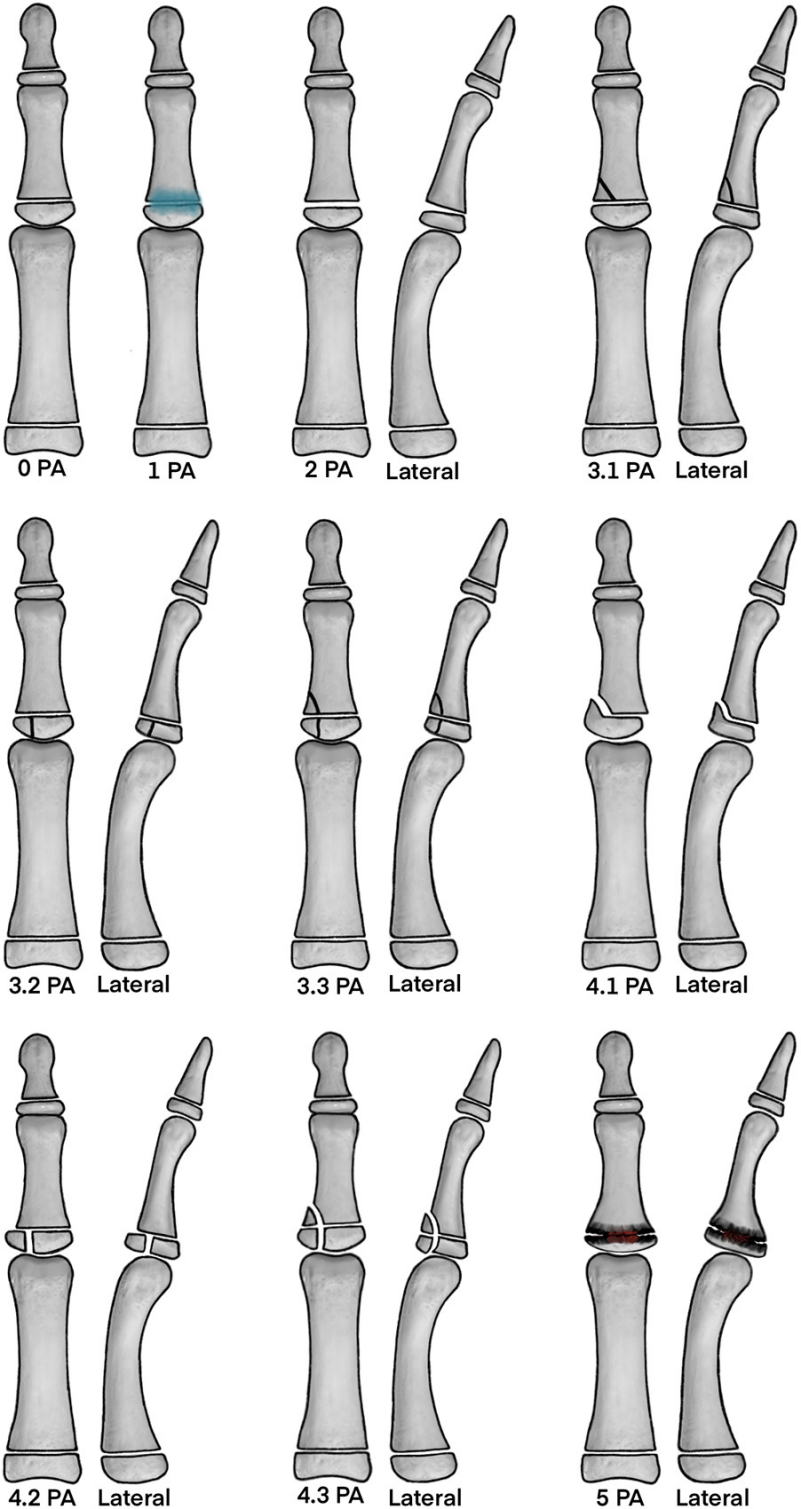
Proposed classification. The illustrations demonstrate the main findings in either x-ray, MRI or CT-scan of the proposed classification. Note that edema may also be present in all higher graded injuries and adjunct to these images the possible condition of sclerosis is defined with an “a” or “b” (a = without sclerosis in CT, b = with sclerosis in CT) (Figure by Nelson T, DC, MS, CSCS, Camp4 Human Performance).

**Table:** 

**Grade**	**Sub-grade**	**Clinical signs**	**Radiological Imaging**
**Grade 0**		Pain during/after climbing. No tenderness over the dorsal distal phalanx on examination.	Normal findings on MRI, US, radiographs or CT. No abnormality of the *“*epiphyseal proliferative zone”.
**Grade 1**		Pain during/after climbing. Tenderness over the dorsal distal phalanx on examination.	Edema present on MRI. No fracture on MRI, US, radiographs or CT. No involvement of the “epiphyseal proliferative zone”.
**Grade 2**		Pain during/after climbing. Tenderness over the dorsal distal phalanx on examination.	Widening at the dorsal middle phalangeal physis on MRI (radiographic sign #1), US, radiographs or CT +/- edema on MRI.
**Grade 3**		Pain during/after climbing. Tenderness over the dorsal distal phalanx on examination.	Nondisplaced fracture on MRI, US, radiographs or CT (radiographic sign #2), +/- dorsal widening of the middle phalangeal physis (radiographic sign #1), +/- edema on MRI.
	Grade 3.1. (a or b)	Pain during/after climbing. Tenderness over the dorsal distal phalanx on examination.	Physeal fracture with metaphyseal extension (analogous to S-H II). (a= without sclerosis in CT, b = with sclerosis in CT)
	Grade 3.2. (a or b)	Pain during/after climbing. Tenderness over the dorsal distal phalanx on examination.	Physeal fracture with epiphyseal extension (analogous to S-H III). (a= without sclerosis in CT, b = with sclerosis in CT)
	Grade 3.3. (a or b)	Pain during/after climbing. Tenderness over the dorsal distal phalanx on examination.	Physeal fracture with epiphyseal and metaphyseal extension (analogous to S-H IV). (a= without sclerosis in CT, b = with sclerosis in CT)
**Grade 4**		Pain during/after climbing. Tenderness over the dorsal distal phalanx on examination +/- palpable osseous fragment overlying the dorsal distal phalanx.	Displaced fracture on MRI, US, radiographs or CT (radiographic sign #3), +/- dorsal widening of the middle phalangeal physis (radiographic sign #1).
	Grade 4.1. (a or b)	Pain during/after climbing. Tenderness over the dorsal distal phalanx on examination +/- palpable osseous fragment overlying the dorsal distal phalanx.	Displaced physeal fracture with metaphyseal extension (analogous to S-H II). (a= without sclerosis in CT, b = with sclerosis in CT)
	Grade 4.2. (a or b)	Pain during/after climbing. Tenderness over the dorsal distal phalanx on examination +/- palpable osseous fragment overlying the dorsal distal phalanx.	Displaced physeal fracture with epiphyseal extension (analogous to S-H III). (a= without sclerosis in CT, b = with sclerosis in CT)
	Grade 4.3. (a or b)	Pain during/after climbing. Tenderness over the dorsal distal phalanx on examination +/- palpable osseous fragment overlying the dorsal distal phalanx.	Displaced fracture with epiphyseal and metaphyseal extension (analogous to S-H IV). (a= without sclerosis in CT, b = with sclerosis in CT)
**Grade 5**		Pain during/after climbing. Tenderness over the dorsal distal phalanx on examination.	“Crush” physeal injury on MRI, US, radiographs or CT (analogous to S-H V). +/- edema on MRI.

## Conclusion

The classification presented is to be considered a proposal pending further evaluation. It is based on scientific analysis, but also personal experience, thus a certain level of bias is possible. Nevertheless, we tried to minimize this by including a radiologist with extensive experience of these injuries, as well as another clinician, in the research team.

Further research is necessary to evaluate the reliability of this classification system and its inter-observer agreement, which we plan to address in future studies.

## Data Availability

The data analyzed in this study is subject to the following licenses/restrictions: xray analysis from former studies were included these studies are published. Requests to access these datasets should be directed to volker.schoeffl@me.com.
